# Effectiveness of Low-Dose Continuous Gaseous Ozone in Controlling *Listeria innocua* on Red Delicious Apples During 9-Month Commercial Cold Storage

**DOI:** 10.3389/fmicb.2021.712757

**Published:** 2021-09-29

**Authors:** Xiaoye Shen, Yuan Su, Zi Hua, Lina Sheng, Manoella Mendoza, Yang He, Tonia Green, Ines Hanrahan, Rob Blakey, Mei-Jun Zhu

**Affiliations:** ^1^School of Food Science, Washington State University, Pullman, WA, United States; ^2^Washington Tree Fruit Research Commission, Wenatchee, WA, United States; ^3^Stemilt Growers LLC., Wenatchee, WA, United States

**Keywords:** Red Delicious apple, *Listeria innocua*, 1-methylcyclopropene, gaseous ozone, quality

## Abstract

This study aimed to investigate the effects of low-dose continuous ozone gas in controlling *Listeria innocua* and quality attributes and disorders of Red Delicious apples during long-term commercial cold storage. Red Delicious apples were inoculated with a three-strain *L. innocua* cocktail at ∼6.2 log_10_ CFU/apple, treated with or without 1-methylcyclopropene, and then subjected to controlled atmosphere (CA) storage with or without continuous gaseous ozone in a commercial facility for 36 weeks. Uninoculated Red Delicious apples subjected to the above storage conditions were used for yeast/mold counts and quality attributes evaluation. The 36 weeks of refrigerated air (RA) or CA storage caused ∼2.2 log_10_ CFU/apple reduction of *L. innocua*. Ozone gas application caused an additional > 3 log_10_ CFU/apple reduction of *L. innocua* compared to RA and CA storage alone. During the 36-week CA storage, low-dose continuous gaseous ozone application significantly retarded the growth of yeast/mold, delayed apple firmness loss, and had no negative influence on ozone burn, lenticel decay, russet, CO_2_ damage, superficial scald, and soft scald of Red Delicious apples compared to CA-alone storage. In summary, the application of continuous low-dose gaseous ozone has the potential to control *Listeria* on Red Delicious apples without negatively influencing apple quality attributes.

## Introduction

*Listeria monocytogenes* can cause deadly listeriosis in susceptible human populations and has a high mortality rate (CDC, 2017). *L. monocytogenes* is widespread in agricultural environments. The Food and Drug Administration enforces a zero tolerance for apples and other ready-to-eat foods (Shank et al., 1996; FDA, 2008). Apples are grown in an open environment and can therefore be subject to *L. monocytogenes* during production or subsequent postharvest packing (Angelo et al., 2017). *L. monocytogenes* has been implicated in two multistate caramel apple outbreaks (Angelo et al., 2017; Marus et al., 2019) and multiple recalls of several fresh apple varieties (FDA, 2017, 2019) and fresh-cut apples (FDA, 2016, 2020b). These outbreaks and recalls highlight the risk of *L. monocytogenes* contamination of apples and the importance of controlling *L. monocytogenes* on apples. After harvest, apples are usually subjected to cold storage for up to 12 months in controlled atmosphere (CA) refrigerated storage. Antimicrobial interventions during this long-term storage could provide potential interventional strategies in controlling *Listeria* on fresh apples (Sheng and Zhu, 2021).

Ozone is a strong oxidant and potent antimicrobial agent, which does not leave any residue on the treated produce (Guzel-Seydim et al., 2004). Ozone gas is approved by the Food and Drug Administration as a Generally Recognized as Safe agent (FDA, 2020a) and is approved for use in organic production and handling (USDA-NOP, 2021). Gaseous ozone at a fixed rate for a short duration has been investigated to control major food-borne pathogens, including *Escherichia coli* O157:H7 (Han et al., 2002), *Salmonella* (Das et al., 2006), and *L. monocytogenes* (Alwi and Ali, 2014; Concha-Meyer et al., 2014) on fresh produce. For example, ozone gas at 10 mg/L resulted in a ∼7 log_10_ CFU/tomato reduction of *Salmonella* Enteritidis inoculated on tomatoes within 1 h (Das et al., 2006). Gaseous ozone at 4 mg/L reduced *L. monocytogenes* by 3 log_10_ CFU/ml on fresh blueberries after 10-day storage at 4°C (Concha-Meyer et al., 2014). A 20-min gaseous ozone (23 mg/L) exposure caused 2.1–3.1 log_10_ CFU/apple reduction of *L. monocytogenes* on Empire apples (Murray et al., 2018). Ozone gas was also studied to control resident microbiota on different apple varieties during storage (Yaseen et al., 2015). A 60-day exposure of ozone gas at 0.5 mg/L under 1°C storage resulted in a 2.7–4.0 log reduction of *Penicillium expansum* on fresh apples, depending on the variety (Yaseen et al., 2015). Furthermore, ozone gas application during storage is beneficial to maintain fruit quality. For example, 1–3 mg/L gaseous ozone exposure was reported to maintain the firmness of apples (Antos et al., 2018) and reduced apple weight loss (Juhnevica-Radenkova et al., 2019). We previously showed that 30 weeks of continuous application of gaseous ozone at 87 μg/L in CA storage reduced *Listeria* by ∼5.0 log_10_ CFU/apple on Fuji apples without negatively impacting apple fruit quality (Sheng et al., 2018). However, this effect cannot directly transfer to other apple cultivars because each apple variety has its unique cuticular wax and resident microbiota compositions that impact *Listeria* persistence and response to antimicrobial interventions (Chai et al., 2020; Ku et al., 2020; Abdelfattah et al., in press).

Red Delicious apples, one of the most commercially sold apple cultivars in the United States (USAA, 2019), was recently implicated in a recall due to potential contamination of *L. monocytogenes* (FDA, 2019). Red Delicious apples are especially prone to developing watercore compared to other commercially grown apple varieties, possessing an increased risk of developing internal disorders such as internal browning during long-term CA storage (Mattheis, 2008), further leading to losses to the apple industry. The objective of this study was to investigate the effects of different commercial storage regimes in conjunction with different doses of continuous gaseous ozone in controlling *Listeria innocua*, which is phylogenetically related to *L. monocytogenes* (Buchrieser et al., 2003), on Red Delicious apples with or without 1-methylcyclopropene (1-MCP) pretreatment. The maintenance of quality attributes of Red Delicious apples under different storage conditions was further evaluated.

## Materials and Methods

### *Listeria innocua* Culture Preparation

Two *L. innocua* food isolates, NRRL 33314 and NRRL 33554, and one processing plant isolate *L. innocua*, NRRL 33197, were obtained from the USDA-ARS culture collection [National Center for Agricultural Utilization Research (NRRL), Peoria, IL, United States] and stored in trypticase soy broth (Becton, Dickinson and Company (BD), Sparks, MD, United States) supplemented with 0.6% yeast extract (TSBYE; Fisher Scientific, Fair Lawn, NJ, United States) (TSBYE) and 20% (v/v) glycerol at −80°C. Each frozen culture was subjected to two sequential transfers in TSBYE at 37°C for 24 h. The three-strain cocktail inoculum was prepared by combining an equal population of individual *L. innocua* strains. The individual *L. innocua* strain or a three-strain cocktail was enumerated by serially diluting and plating on TSAYE (TSBYE with 1.5% agar) plates.

### Apple Inoculation

Fresh unwaxed Red Delicious apples, manually harvested at commercial maturity as indicated by the Cornell Starch index for a range of 2.8–3.5 (Blanpied and Silsby, 1992), were stored in a commercial storage room (Yakima, WA, United States) at ∼1°C and delivered to the laboratory before the study. Apples (∼200 g), free of cuts, bruises, or scars, were selected for this study. For *L. innocua* inoculation, apples were dip inoculated in the three-strain *L. innocua* inoculum diluted in sterile phosphate-buffered saline (1 × PBS, pH 7.4) to obtain the inoculation level of ∼6.2 log_10_ CFU/apple. The inoculated apples were held at room temperature (∼22°C) for 24 h before subjected to the respective storages. Forty inoculated apples were randomly sampled at 0 and 24 h, respectively, to confirm the initial bacterial level and the uniformity of inoculation.

### Storage Treatment of Apples

Apples 24-h postinoculation were randomly packed into plastic crates; 40 apples per crate. The boxed inoculated apples were randomly separated into six groups, and half of them were treated with 1.0 mg/L gaseous 1-MCP air for 24 h before storage. Apples were then subjected to the refrigerated air (RA; 0.2 ± 0.1°C) and CA (0.2 ± 0.1°C, 3.1 ± 0.0% O_2_, 0.4 ± 0.0% CO_2_) with or without continuous gas at 60.2 ± 5.7 or 78.7 ± 13.2 μg/L in semicommercial RA/CA rooms (3,200 ft^2^) with the relative humidity of ∼90% in a commercial facility (Stemilt Growers LLC, Wenatchee, WA, United States). Ozone gas was generated by a commercial ozone generator (Guardian Ozone, Cocoa, FL, United States) and transferred automatically into storage room coupled with a proportional–integral–derivative algorithm to maintain ozone dose at the target level during storage. Ozone concentrations were initiated at the first week of storage and reached the target concentration at the beginning of the fourth week. Concurrently, separated sets of uninoculated apples were included in the above storage conditions for resident microflora enumeration and apple quality evaluation.

Inoculated apples were sampled after 3, 6, 12, 18, 24, 30, and 36 weeks of storage for *L. innocua* enumeration. Uninoculated apples were sampled after 6, 12, 24, and 36 weeks of storage for total plate counts (TPC) and yeast/mold counts. Apple quality attributes were assessed at harvest and at 6 and 9 months of storage. Forty apples per storage regime were sampled at each time point for microbial enumeration, quality attributes, and internal disorder evaluation, while 100 apples per storage regime were sampled at each time point for external disorder evaluation, where each apple was considered an experimental unit.

### *Listeria innocua* Enumeration

Each apple was transferred to a stomacher bag (Fisher Scientific) containing 10 ml of sterile 1 × PBS and hand-rubbed for 80 s. Rub solutions were 10-fold serially diluted with sterile 1 × PBS, plated on TSAYE plates, and then overlaid with modified Oxford agar (MOX) (BD) to discern *Listeria* from background bacteria (Shen et al., 2019), and incubated at 37°C for 48 h. *L. innocua* produced typical black colonies surrounded by black halos on MOX agar plates. For samples below the detection limit (10 CFU/apple), 1.0 ml of rub solution was enriched in buffered *Listeria* enrichment buffer (BLEB) (BD) at 30°C for 48 h; the presence/absence of *Listeria* was reported. The enrichment culture was streaked onto both MOX and CHROMagar^TM^ *Listeria* (CHROMagar, Paris, France) plates, respectively, to qualitatively detect the presence of *Listeria* (Sheng et al., 2017). A sample size of 10 apples per replication with four independent replicates per storage regime was sampled at each time point.

### Resident Microbiota Enumeration

Uninoculated apples were processed the same way as inoculated apples. Rub solutions at appropriate dilutions were plated on duplicate TSAYE plates and potato dextrose agar (PDA; BD) plates, respectively, for TPC and yeast/mold counts. TSAYE plates were incubated at 37°C for 24 h, while PDA plates were incubated at room temperature (22°C) for 5 days. A sample size of 10 apples per replication with four independent replicates per storage regime was sampled at each time point.

### Fruit Quality Analysis

Fruit quality attributes including firmness, total soluble solids (TSS), and titratable acidity (TA) were evaluated on uninoculated apples at harvest and at 6 and 9 months after cold storage per the published method (Sheng et al., 2018). Briefly, fruit firmness was measured with a fruit texture analyzer (FTA GS-15-643, Güss Manufacturing, Ltd., Strand, South Africa) using a 1-cm-diameter probe on a peeled area (∼3 cm^2^) on both the sun-exposed and shaded sides of each apple. TSS content was measured using an Atago PR-32 digital Brix refractometer (Atago Co., Ltd., Tokyo, Japan). TA of fruit juice was represented by the percentage of malic acid content (grams of malic acid per 100 g fresh weight of apples) and measured using a potentiometric titrator (Titrando 888 and 875 Robotic USB Sample Processor XL, Metrohm, Riverview, FL, United States). A sample size of 10 apples per replication with four independent replicates per storage regime was used for apple quality analysis.

### Fruit Disorder Analysis

Fruits were inspected for external disorders at harvest and after 6 and 9 months of cold storage. External disorders including ozone burn (surface pitting on apple exposed side), superficial scald (brown patches and discoloration on the skin), lenticel decay (dark brown pits around lenticels), decay (visible mycelium or spore masses), russet (periderm formation on skin), and CO_2_ damage (snowflake-like patches on the skin) were visually inspected following standard criteria as previously described (Sheng et al., 2018). Soft scald, characterized by smooth or irregularly shaped brown lesions on apple surfaces (DeEll, 2013), was also evaluated. Apples were sliced three times for evaluation of internal disorders including watercore and internal browning. Watercore was characterized by the accumulation of sorbitol-rich fluid in the intercellular spaces in flesh adjacent to the vasculature (Marlow and Loescher, 1984). Internal browning was characterized by the brown discoloration of the flesh that usually originated near the core area (Argenta et al., 2001). The results were reported as apples with respective disorder/total apples (%) within individual treatment per storage period. A sample size of 10 apples per replicate with four independent replicates per storage regimen was used for internal disorder assessment. For external disorder evaluations, a sample size of 100 apples was used per each storage regime. Apples were evaluated after 1 and 7 days at room temperature (22°C).

### Statistical Analysis

Data were analyzed with IBM SPSS 19.0 (Chicago, IL, United States). Mean difference was discerned by one-way analysis of variance (ANOVA) followed by Tukey multiple comparison test. Values of *p* < 0.05 were considered statistically significant.

## Results

### Survival of *Listeria innocua* on Red Delicious Apples Under Commercial Cold Storage Conditions With Different Doses of Gaseous Ozone

**FIGURE 1:**
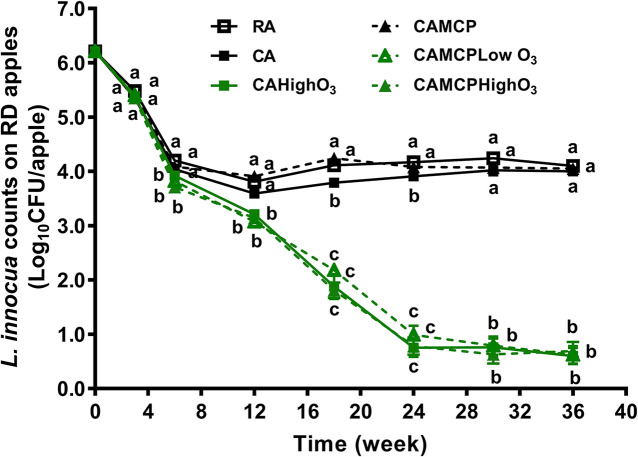
Survival of *L. innocua* on Red Delicious apples during 36 weeks of commercial cold storage. ^a– c^Mean at each sampling point without a common letter differ significantly (*p* < 0.05). Mean ± SEM, *n* = 32–40. MCP, apples were treated with 1-MCP prior to cold storage; CAHighO_3_, CA storage with continuous gaseous O_3_ application at 78.7 ± 13.2 μg/L; CAMCPHighO_3_, CA storage with continuous gaseous O_3_ application at 78.7 ± 13.2 μg/L, where apples were treated with 1-MCP prior to cold storage; CAMCPLowO_3_, CA storage with continuous gaseous O_3_ application at 60.2 ± 5.7 μg/L, where apples were treated with 1-MCP prior to cold storage.

During the first 3 weeks of cold storage, the populations of *L. innocua* on apples were reduced by 0.7–0.9 log_10_ CFU/apple under all storage conditions, before the ozone concentration had reached the target concentration (Figure 1). There was 2.1–2.2 log_10_ CFU/apple reduction of *L. innocua* on Red Delicious apples under RA storage or CA storage with or without 1-MCP treatment during 36 weeks of storage (Figure 1). Low-dose continuous gaseous ozone application, regardless of dose and 1-MCP treatment, dramatically increased *L. innocua* reduction on Red Delicious apples compared to that under RA or CA storage, which resulted in an additional 3.3–3.5 log_10_ CFU/apple reduction at the end of 24 weeks of storage followed by no further reduction during subsequent storage (Figure 1 and Table 1).

**TABLE 1:** *Listeria innocua-*positive Red Delicious apples under CA with gaseous ozone and/or 1-MCP storage after 18 weeks.

Sampling point	Treatment	Enumeration (log_10_ CFU/Apple)	Plating positive	Enrichment positive
18 weeks	CAMCPLowO_3_	2.2 ± 0.1^a^	40/40	40/40
	CAMCPHighO_3_	1.8 ± 0.1^a^	34/40	39/40
	CAHighO_3_	2.0 ± 0.2^a^	30/40	36/40
24 weeks	CAMCPLowO_3_	1.0 ± 0.2^a^	20/40	25/40
	CAMCPHighO_3_	0.8 ± 0.2^a^	18/40	24/40
	CAHighO_3_	0.8 ± 0.2^a^	20/40	23/40
30 weeks	CAMCPLowO_3_	0.8 ± 0.2^a^	15/40	16/40
	CAMCPHighO_3_	0.6 ± 0.2^a^	11/40	15/40
	CAHighO_3_	0.8 ± 0.2^a^	15/40	17/40
36 weeks	CAMCPLowO_3_	0.6 ± 0.2^a^	12/40	18/40
	CAMCPHighO_3_	0.7 ± 0.2^a^	13/40	20/40
	CAHighO_3_	0.6 ± 0.2^a^	13/40	18/40

*Plating positive or enrichment positive was represented as apples positive for *L. innocua* plating or enrichment/total apples tested.*

*CAHighO_3_, CA storage with continuous gaseous O_3_ application at 78.7 ± 13.2 μg/L; CAMCPHighO_3_, CA storage with continuous gaseous O_3_ application at 78.7 ± 13.2 μg/L, where apples were treated with 1-MCP prior to cold storage; CAMCPLowO_3_, CA storage with continuous gaseous O_3_ application at 60.2 ± 5.7 μg/L, where apples were treated with 1-MCP prior to cold storage.*

**n* = 40.*

*^*a*^Mean within a column at each sampling point with no common letter differ significantly (*p* < 0.05).*

### Resident Microflora on Red Delicious Apples Under Commercial Cold Storage Conditions With Different Doses of Gaseous Ozone

**FIGURE 2:**
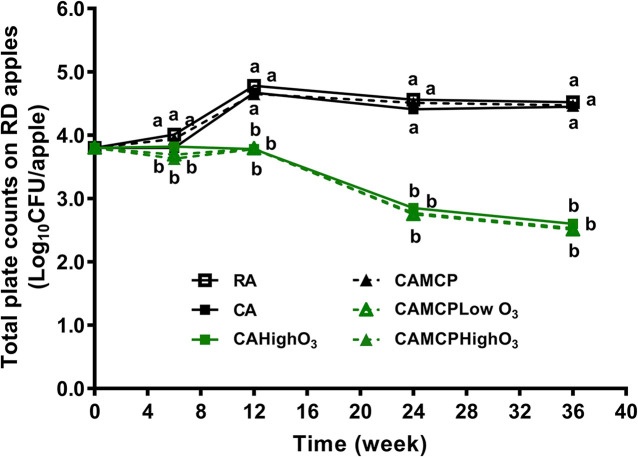
Resident bacteria on Red Delicious apples during 36 weeks of commercial cold storage. ^a,b^Mean at each sampling point without common letter differ significantly (*p* < 0.05). Mean ± SEM, *n* = 40. MCP, apples were treated with 1-MCP prior to cold storage; CAHighO_3_, CA storage with continuous gaseous O_3_ application at 78.7 ± 13.2 μg/L; CAMCPHighO_3_, CA storage with continuous gaseous O_3_ application at 78.7 ± 13.2 μg/L, where apples were treated with 1-MCP prior to cold storage; CAMCPLowO_3_, CA storage with continuous gaseous O_3_ application at 60.2 ± 5.7 μg/L, where apples were treated with 1-MCP prior to cold storage.

The resident bacteria on uninoculated Red Delicious apples were 3.8 log_10_ CFU/apple before storage (Figure 2). The population of resident bacteria on apples increased by 0.9–1.0 log_10_ CFU/apple under RA or CA storage pretreated with or without 1-MCP (Figure 2). The level of resident bacteria was relatively stable during subsequent RA and CA storage (Figure 2). Gaseous ozone application decreased resident bacteria by 1.2–1.3 log_10_ CFU/apple after 36 weeks of storage regardless of ozone dose and 1-MCP pretreatment (Figure 2).

**FIGURE 3:**
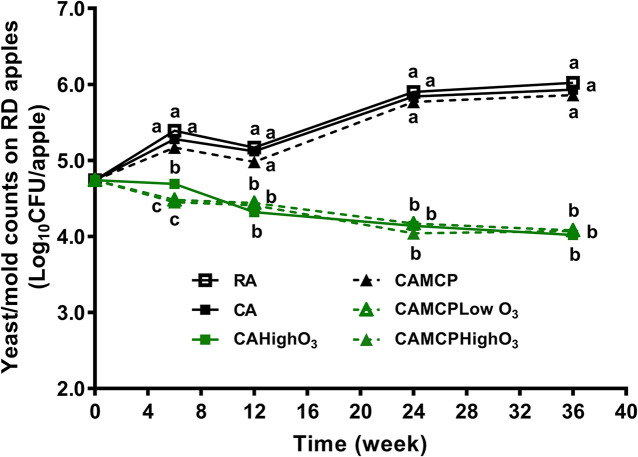
Yeast/mold counts on Red Delicious apples during 36 weeks of commercial cold storage. ^a– c^Mean at each sampling point without common letter differ significantly (*p* < 0.05). Mean ± SEM, *n* = 40. MCP, apples were treated with 1-MCP prior to cold storage; CAHighO_3_, CA storage with continuous gaseous O_3_ application at 78.7 ± 13.2 μg/L; CAMCPHighO_3_, CA storage with continuous gaseous O_3_ application at 78.7 ± 13.2 μg/L, where apples were treated with 1-MCP prior to cold storage; CAMCPLowO_3_, CA storage with continuous gaseous O_3_ application at 60.2 ± 5.7 μg/L, where apples were treated with 1-MCP prior to cold storage.

The yeast/mold counts of uninoculated Red Delicious apples were 4.7 log_10_ CFU/apple at the beginning of storage, which increased by 1.1–1.3 log_10_ CFU/apple after 36 weeks of RA or CA storage (Figure 3). The gaseous ozone application, regardless of dose and 1-MCP treatment, resulted in a 0.7 log_10_ CFU/apple reduction of yeast/mold counts after 36 weeks of storage (Figure 3).

### Quality Attributes and Disorders of Red Delicious Apples Under Commercial Cold Storage Conditions With Different Doses of Gaseous Ozone

The TSS of Red Delicious apples did not differ among storage treatments at 6 and 9 months (Table 2). Red Delicious apples under RA storage had a significantly lower firmness and TA compared with CA with 1-MCP treatment or with gaseous ozone at 6- and 9-month storages (Table 2). Ozone gas application or 1-MCP treatment in CA storage significantly retarded fruit firmness loss compared to CA storage alone at 6 and 9 months of storage, where the firmness was 4.4 and 4.2 kg for CA storage but was 5.5–6.5 and 5.2–6.8 kg for CA storage with MCP treatment and ozone gas application (Table 2).

**TABLE 2:** Fruit quality attributes of Red Delicious apples after cold storage under different conditions.

Treatment	Firmness (kg)			TSS (% Brix)			TA (% malic acid)^1^		
			
	At harvest	6-month	9-month	At harvest	6-month	9-month	At harvest	6-month	9-month
RA	6.6 ± 0.2^A^	3.8 ± 0.1^aB^	3.9 ± 0.1^aB^	12.9 ± 0.2^A^	12.5 ± 0.2^aA^	12.5 ± 0.2^aA^	0.3 ± 0.0^A^	0.1 ± 0.0^aB^	0.1 ± 0.0^aB^
CA		4.4 ± 0.3^aB^	4.2 ± 0.1^aB^		14.2 ± 0.3^bA^	13.2 ± 0.2^abA^		0.2 ± 0.0^abB^	0.2 ± 0.0^abB^
CAMCP		6.3 ± 0.2^bcA^	5.8 ± 0.2^bcA^		13.0 ± 0.8^abA^	13.8 ± 0.1^abA^		0.1 ± 0.0^abB^	0.2 ± 0.0^bB^
CAMCPLowO_3_		6.5 ± 0.2^bcA^	5.7 ± 0.2^bcA^		14.0 ± 0.4^abA^	13.9 ± 0.2^abA^		0.2 ± 0.0^bB^	0.2 ± 0.0^bB^
CAMCPHighO_3_		6.5 ± 0.2^bcA^	6.8 ± 0.1^bA^		13.7 ± 0.1^abA^	14.0 ± 0.2^bA^		0.2 ± 0.0^bB^	0.2 ± 0.0^bB^
CAHighO_3_		5.5 ± 0.2^cB^	5.2 ± 0.2^cB^		14.0 ± 0.2^bA^	13.4 ± 0.1^abA^		0.2 ± 0.0^bB^	0.2 ± 0.0^bB^

*MCP, apples were treated with 1-MCP prior to cold storage; CAHighO_3_, CA storage with continuous gaseous O_3_ application at 78.7 ± 13.2 μg/L; CAMCPHighO_3_, CA storage with continuous gaseous O_3_ application at 78.7 ± 13.2 μg/L, where apples were treated with 1-MCP prior to cold storage; CAMCPLowO_3_, CA storage with continuous gaseous O_3_ application at 60.2 ± 5.7 μg/L, where apples were treated with 1-MCP prior to cold storage.*

*^1^Percentage of malic acid were reported by grams of malic acid per 100-g fresh weight of apples.*

*^*a*–*c*^Mean within a column without common letter differ significantly (*p* < 0.05).*

*^*A,B*^Mean of the comparison of individual quality parameter at harvest, 6-month, and 9-month storage within each storage treatment without common letter differ significantly (*p* < 0.05).*

*Mean ± SEM, *n* = 40.*

Red Delicious apples under RA storage had a higher incidence of superficial scald (at 6- and 9-month of storage) and rot (at 9-month of storage) compared to CA storage with or without 1-MCP treatment (Table 3). Compared to CA storage, gaseous ozone application in CA storage did not influence the incidence of superficial scald, ozone burn, lenticel decay, russet, CO_2_ damage, and soft scald of Red Delicious apples up to 9 months of storage (Table 3).

**TABLE 3:** External disorders analysis of Red Delicious apples after cold storage under different conditions^1^.

Treatment	Ozone burn	Superficial scald	Lenticel decay	Rot	Russet	CO_2_ damage	Soft scald
At harvest	0^A^	0^A^	0^A^	0^A^	0^A^	0^A^	0^A^
**6-month**							
RA	0^aA^	14.2 ± 0.1^aB^	0^aA^	1.0 ± 0.0^aA^	0^aA^	0^aA^	0^aA^
CA	0^aA^	0^bA^	0^aA^	0^aA^	0^aA^	0.5 ± 0.0^aA^	2.6 ± 0.0^aA^
CAMCP	0^aA^	2.0 ± 0.0^bA^	0^aA^	1.0 ± 0.0^aA^	1.0 ± 0.0^aA^	1.0 ± 0.0^aA^	0^aA^
CAMCPLowO_3_	0^aA^	1.0 ± 0.0^bA^	0^aA^	2.0 ± 0.0^aA^	0^aA^	0^aA^	0.5 ± 0.0^aA^
CAMCPHighO_3_	0^aA^	0^bA^	0^aA^	0^aA^	1.0 ± 0.0^aA^	1.0 ± 0.0^aA^	0.5 ± 0.0^aA^
CAHighO_3_	0^aA^	0^bA^	0^aA^	0^aA^	4.8 ± 0.0^bB^	0^aA^	1.0 ± 0.0^aA^
**9-month**							
RA	0^aA^	38.0 ± 0.1^aB^	0^aA^	24.4 ± 0.1^aB^	0^aA^	0^aA^	0^aA^
CA	0^aA^	7.5 ± 0.0^bA^	3.0 ± 0.0^aA^	4.5 ± 0.0^bA^	2.0 ± 0.0^abA^	0^aA^	0^aA^
CAMCP	0^aA^	1.0 ± 0.0^bA^	1.0 ± 0.0^aA^	2.1 ± 0.0^bA^	2.1 ± 0.0^abA^	0^aA^	0^aA^
CAMCPLowO_3_	0^aA^	2.0 ± 0.0^bA^	3.0 ± 0.0^aA^	1.0 ± 0.0^bA^	4.0 ± 0.0^bB^	1.0 ± 0.0^aA^	0^aA^
CAMCPHighO_3_	0^aA^	6.6 ± 0.0^bA^	3.8 ± 0.0^aA^	2.7 ± 0.0^bA^	2.7 ± 0.0^abA^	0^aA^	0^aA^
CAHighO_3_	0^aA^	5.5 ± 0.0^bA^	4.0 ± 0.0^aA^	0.4 ± 0.0^bA^	1.8 ± 0.0^abA^	0^aA^	0^aA^

*^1^Data were presented as percentage (%).*

*MCP, apples were treated with 1-MCP prior to cold storage; CAHighO_3_, CA storage with continuous gaseous O_3_ application at 78.7 ± 13.2 μg/L; CAMCPHighO_3_, CA storage with continuous gaseous O_3_ application at 78.7 ± 13.2 μg/L, where apples were treated with 1-MCP prior to cold storage; CAMCPLowO_3_, CA storage with continuous gaseous O_3_ application at 60.2 ± 5.7 μg/L, where apples were treated with 1-MCP prior to cold storage.*

*^*a,b*^Mean within a column without common letter differ significantly for 6- or 9-month storage (*p* < 0.05).*

*^*A,B*^Mean within a column, each external quality parameter was compared through at harvest, 6-month, and 9-month storage, and without common letter differ significantly (*p* < 0.05).*

*Mean ± SEM, *n* = 100, averaged from 1- and 7-day ripening after storage at room temperature.*

The incidence of watercore was decreased in Red Delicious apples under all storage treatments after 6- and 9-month of storage (Table 4). The internal browning incidence was significantly increased in Red Delicious apples under RA and CA storage, with or without 1-MCP at 6 and 9 months, compared to that at harvest (Table 4). The application of gaseous ozone at 60.2–78.7 μg/L during CA storage mitigated the internal browning incidence of Red Delicious apples (Table 4).

**TABLE 4:** Internal disorder analysis of Red Delicious apples after cold storage under different conditions^1^.

Treatment	Watercore			Internal browning		
		
	At harvest	6-month	9-month	At harvest	6-month	9-month
RA	74.4 ± 1.1^A^	5.0 ± 0.0^aB^	0^aB^	0^A^	47.5 ± 0.1^aB^	10.0 ± 0.0^acA^
CA		0^aB^	0^aB^		50.0 ± 0.1^aB^	40.0 ± 0.0^bB^
CAMCP		0^aB^	0^aB^		37.5 ± 0.1^abB^	27.5 ± 0.0^bcB^
CAMCPLowO_3_		0^aB^	0^aB^		20.0 ± 0.0^bA^	15.0 ± 0.1^acA^
CAMCPHighO_3_		0^aB^	0^aB^		15.0 ± 0.1^bA^	2.5 ± 0.0^acA^
CAHighO_3_		0^aB^	0^aB^		25.0 ± 0.1^abA^	12.5 ± 0.0^cA^

*^1^Data were presented as percentage (%).*

*MCP, apples were treated with 1-MCP prior to cold storage; CAHighO_3_, CA storage with continuous gaseous O_3_ application at 78.7 ± 13.2 μg/L; CAMCPHighO_3_, CA storage with continuous gaseous O_3_ application at 78.7 ± 13.2 μg/L, where apples were treated with 1-MCP prior to cold storage; CAMCPLowO_3_, CA storage with continuous gaseous O_3_ application at 60.2 ± 5.7 μg/L, where apples were treated with 1-MCP prior to cold storage.*

*^*a*–*c*^Mean within a column without common letter differ significantly (*p* < 0.05).*

*^*A,B*^Mean of the comparison of individual internal disorder parameter at harvest, 6-month, and 9-month storage within each storage treatment without common letter differ significantly (*p* < 0.05).*

*Mean ± SEM, *n* = 40.*

## Discussion

Red Delicious apples at 36 weeks of RA and CA commercial cold storages exhibited a 2.1–2.2 log_10_ CFU/apple reduction of *L. innocua*. Similarly, a 2.5–3.0 log_10_ CFU/apple reduction of *L. innocua* was detected on Fuji apples at 30 weeks of RA and CA cold storages (Sheng et al., 2018). Consistently, a 10-day CA storage at 4°C reduced *L. monocytogenes* by 1.2 log_10_ CFU/ml on fresh highbush blueberries (Concha-Meyer et al., 2014). This study found a similar reduction of *L. innocua* on the surface of Red Delicious apples during 36 weeks of RA and CA storages. However, more reduction of *L. innocua* was observed in Fuji apples under RA storage than that under CA storage (Sheng et al., 2018). *L. monocytogenes* showed better survival on endive under RA storage at 4°C compared to that under CA storage (5% CO_2_, 5%O_2_, 90% N_2_), where an additional 1.0 log reduction of *L. monocytogenes* occurred on endive under CA storage after 14 days (Niemira et al., 2005). The exact reasons for the different behavior of *L. innocua* in different apple varieties/studies are unknown. Different *Listeria* strains are known to have different behavior under 56-day CA (5% O_2_, 10% CO_2_, and 85% N_2_) storage at 4°C (Razavilar and Genigeorgis, 1992). Given that the same *L. innocua* cocktail was used in this study as previous reports using Fuji apples, the resident microbial community associated with each apple variety, the source of apples, and the apple surface wax composition might be partially responsible for the divergence, warranting future research.

The gaseous ozone application at 60.2–78.7 μg/L during 24 weeks of CA storage resulted in > 5 log_10_ CFU/apple reductions of *L. innocua* on Red Delicious apples, which is consistent with the finding on Fuji apples (Sheng et al., 2018). The 1-MCP application, a common practice for apple fruit subjected to long-term CA storage (Mattheis, 2008), did not interfere the antilisterial efficacy of gaseous ozone throughout the entire storage. Considering practical *L. monocytogenes* contamination loads were less than 3.5 log_10_ CFU/fruit (Chen et al., 2016), the gaseous ozone application provides a viable in-storage intervention strategy in controlling *L. monocytogenes* on fresh apples. It is worth noting that the maximal reduction of *L. innocua* was achieved at 24 weeks of CA storage with gaseous ozone; ozone application during subsequent storage did not cause additional reduction of *L. innocua*. A similar phenomenon was also observed in Fuji apples, where lethality of ozone gas against *L. innocua* reached a plateau at 24 weeks of storage (Sheng et al., 2018). A 6-h exposure to 1 mg/L ozone reduced *L. monocytogenes* on fresh-cut bell peppers by 2 log_10_ CFU/g, and prolonged exposure of 24 h did not result in any further reduction (Alwi and Ali, 2014). Gaseous ozone is highly mobile and reactive compared to aqueous ozone intervention on produce surfaces (Singh et al., 2002), and it induces cell leakage by increasing membrane fluidity, reducing membrane integrity, and disrupting cell osmotic balance (Zhang et al., 2011; Ersoy et al., 2019). The *L. innocua* on apple surfaces might be protected from ozone by dead cells or microcrack/niches on the apple surfaces (Macauley et al., 2006; Selma et al., 2008).

The initial populations of resident bacteria and yeast/mold counts on Red Delicious apples were 3.8 and 4.7 log_10_ CFU/apple, which was similar with previous observations on Fuji apples (Sheng et al., 2018) and Red Delicious apples (Kreske et al., 2006), indicating apple surfaces are covered with a high population of background microflora. TPC and yeast/mold counts increased by ∼1 log_10_ CFU/apple on Red Delicious apples under RA and CA storage after 36 weeks. Similarly, TPC and yeast/mold counts increased on sweet cherries under CA storage at 1°C for 30 days (Serradilla et al., 2013). The yeast/mold counts of Fuji apples increased by ∼1 log_10_ CFU/apple during 30 weeks of RA storage (Sheng et al., 2018). In contrast, TPC of Fuji apples under RA and CA storage and the yeast/mold population of Fuji apples under CA remained relatively stable during 30 weeks (Sheng et al., 2018). This difference might be due to different initial background resident microbiota compositions as well as the different surface properties of Fuji and Red Delicious apples.

Ozone gas application at 60.2–78.7 μg/L in CA storage suppressed the resident microflora on Red Delicious apples. This was consistent with the finding on Fuji apples, where supplementation of 87 μg/L gaseous ozone in CA storage retarded the growth of resident bacteria and yeasts and molds on apples at 30 weeks of storage (Sheng et al., 2018). Concordantly, continuous provision of 0.5 mg/L ozone gas during 15 days of storage at 12°C reduced resident microbiota, including total mesophilic bacteria and yeast/mold counts, by ∼0.2 log_10_ CFU/g on tomatoes (Tuffi et al., 2012). A 45-min ozone gas treatment at 2.8 mg/L reduced TPC and yeast/mold counts by 1.5 and 0.7 log_10_ CFU/mushroom on mushrooms (Akata et al., 2015).

Fruit quality is an important driver of consumer preference. Improper cold storage leads to a significant loss of fruit quality (Davis and Blair, 1936). CA storage is known to maintain the fruit quality longer (Tasdelen and Bayindirli, 1998) and retard apple decay during long-term storage (Xuan and Streif, 2005). Consistently, Red Delicious apples under RA storage had a higher incidence of superficial scald and decay compared to CA storage, pretreated with or without 1-MCP treatment, during 36 weeks of storage.

The supplementation of low-dose continuous gaseous ozone in CA storage significantly retarded the firmness loss in Red Delicious apples compared to that in RA or CA storage alone. Similarly, the application of gaseous ozone in CA storage after 24 weeks significantly decreased the firmness loss in Fuji apples compared to that in RA storage (Sheng et al., 2018). However, the changes of apple firmness were similar for Fuji apples under CA storage alone or supplemented with ozone gas (Sheng et al., 2018). Accordingly, the application of gaseous ozone at 0.8 mg/L during 6 months of cold storage retarded the loss of flesh firmness in Ledzenu and Auksis apples (Juhnevica-Radenkova et al., 2019).

The 1-MCP pretreatment was beneficial to maintain the firmness in Red Delicious apples during 9-month CA storage. Consistently, Empire apples pretreated with 1-MCP were firmer than apples without 1-MCP treatment after 10 months of 0.5°C CA storage (Jung and Watkins, 2011); Red Delicious apples treated with 1-MCP were firmer than those without 1-MCP treatment after 6 months of 0°C storage (Mir et al., 2001). 1-MCP is a known ethylene action inhibitor, which binds to ethylene receptor and prevents ethylene from binding and eliciting its action, thus retarding fruit ripening and firmness loss (Deell et al., 2008).

Watercore is a physiological disorder where fluid accumulates in the intercellular air spaces in flesh adjacent to the vasculature (Marlow and Loescher, 1984). Red Delicious and Fuji apples are two cultivars that are prone to developing watercore (Mattheis, 2008). In this study, the incidence of watercore in Red Delicious apples at harvest was high. This might be due to the fact that Red Delicious apples typically, when achieving the optimum background color for commercial harvest requirements, are more likely to have watercore at harvest (Watkins et al., 1992). The incidence of watercore was significantly reduced during cold storage regardless of storage treatment/period. In agreement, the incidence of watercore decreased from 2.6 to 0.1 as expressed by watercore rating in Fuji apples after 160 days of storage at 0°C (Kasai and Arakawa, 2010). In this study, the internal browning developed in Red Delicious apples under all storage conditions after 6- and 9-month storages but was mitigated by supplementing with gaseous ozone in CA storage compared with CA storage alone. In Fuji apples, no internal browning was found after 6 months of RA or CA storage (Sheng et al., 2018). Consistent with our finding on Fuji apples (Sheng et al., 2018), continuous low-dose gaseous ozone application did not cause ozone burn and had no impact on superficial scald, lenticel decay, russet, CO_2_ damage, and soft scald on Red Delicious apples over 9 months of CA storage.

## Conclusion

The counts of *L. innocua* on Red Delicious apples decreased by ∼2 log CFU/apple over 9 months of commercial RA or CA storage. The reduction of *L. innocua* was magnified 1,000-fold by continuous low-dose ozone gas treatment at 60.2–78.7 μg/L; > 5.0 log_10_ CFU/apple reduction was achieved after 24 weeks of storage. In addition, low-dose gaseous ozone application in CA storage retarded the growth of resident microbiota, delayed apple firmness loss, improved apple quality, and had no negative impacts on external disorders of Red Delicious apples compared to CA storage without ozone gas. Thus, low-dose continuous gaseous ozone application is a promising strategy for the apple industry to control *Listeria* and microbial decay on Red Delicious apples. Further studies on different ozone dosage and apple varieties are warranted to ensure the safety and quality of fresh apples.

## Data Availability Statement

The raw data supporting the conclusions of this article will be made available by the authors, without undue reservation.

## Author Contributions

XS: investigation, data analysis, and writing–original draft. YS, ZH, LS, and MM: investigation. YH and TG: sample preparation. IH and RB: writing–review and editing. M-JZ: conceptualization, supervision, data analysis, writing–review and editing, and funding acquisition. All authors contributed to the article and approved the submitted version.

## Conflict of Interest

RB was employed by Stemilt Growers LLC. The remaining authors declare that the research was conducted in the absence of any commercial or financial relationships that could be construed as a potential conflict of interest.

## Publisher’s Note

All claims expressed in this article are solely those of the authors and do not necessarily represent those of their affiliated organizations, or those of the publisher, the editors and the reviewers. Any product that may be evaluated in this article, or claim that may be made by its manufacturer, is not guaranteed or endorsed by the publisher.
